# Generating Publishable Data from Course-Based Undergraduate Research Experiences in Chemistry

**DOI:** 10.1021/acs.jchemed.3c00354

**Published:** 2023-08-10

**Authors:** Amanda L. Wolfe, P. Ryan Steed

**Affiliations:** Department of Chemistry and Biochemistry, University of North Carolina Asheville, Asheville, North Carolina 28804, United States

**Keywords:** Upper-Division Undergraduate, Curriculum, Interdisciplinary/Multidisciplinary, Laboratory Instruction, Inquiry-Based/Discovery Learning, Undergraduate Research

## Abstract

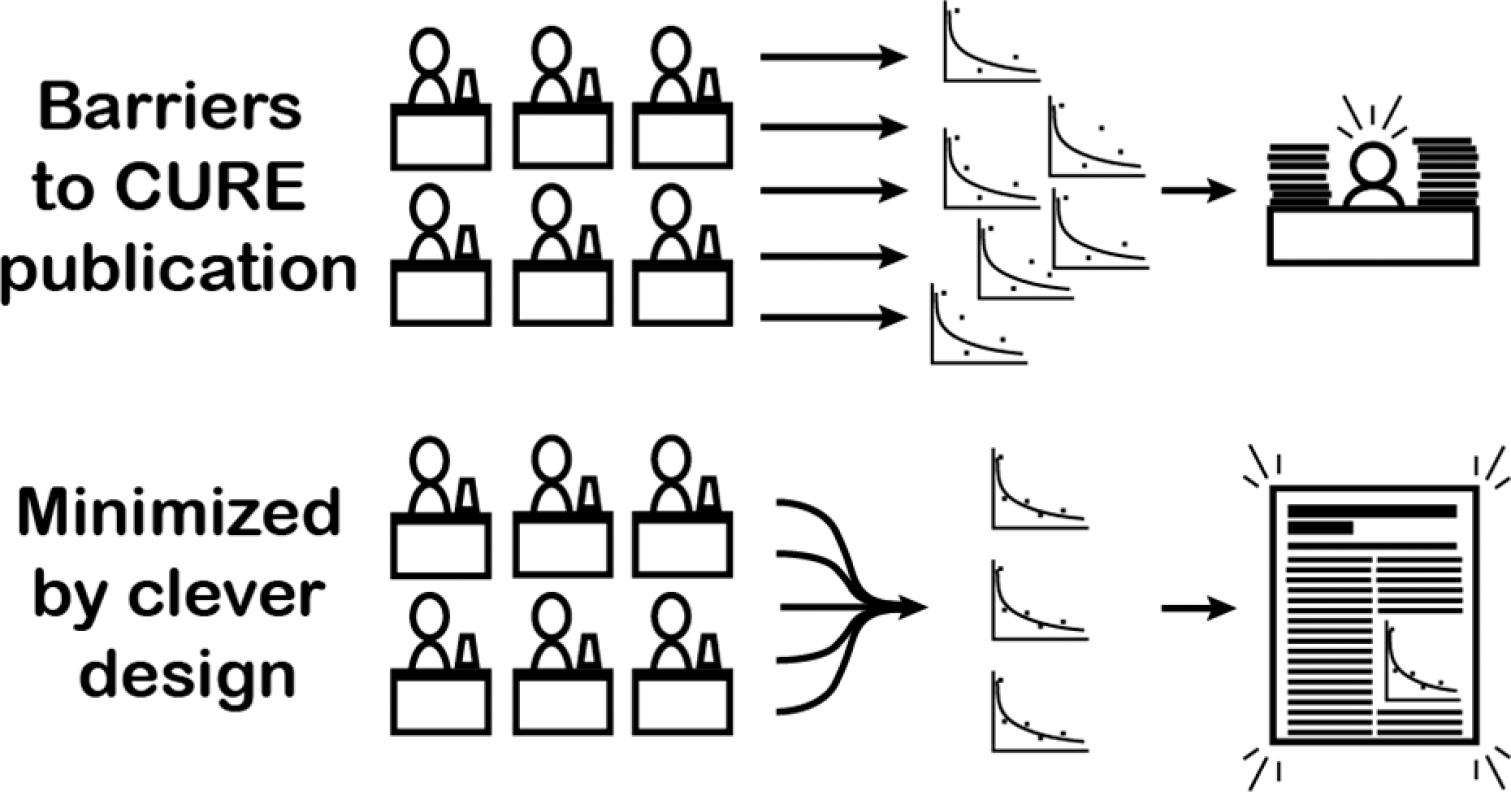

Embedding Course-based Undergraduate Research Experiences (CUREs) into chemistry curricula has become a best practice due to the overwhelming evidence that these experiences deepen students’ content comprehension, improve students’ problem-solving skills, and increase retention within the major. For these reasons, faculty are often encouraged to develop CUREs for their courses, which typically take a substantial amount of effort and administrative/financial support. To justify these efforts, one of the most cited benefits of CURE development for faculty specifically is that they can pilot research projects and publish data produced during CUREs in scientific publications. However, there is less evidence in the literature that these benefits commonly occur. Based on direct upper-level, interdisciplinary CURE development experience and a national survey of faculty across institution types, it is clear that translating CURE data into publishable science is quite challenging due to several common barriers. Barriers identified include the need for follow up data that must be generated by either the faculty or a research student, the lack of reproducibility of data generated by novice students, and the lack of faculty time to write the manuscripts. Additionally, institution type (private vs public non-PhD granting; non-PhD granting vs PhD granting), faculty rank, and CURE level (lower vs upper-level courses), among other factors, impacted the likelihood of publication of CURE data. Based on these results and experiences, best practices for maximizing positive outcomes for both students and faculty with regard to CURE design and implementation have been developed.

Course-based undergraduate research experiences (CUREs) have been infused throughout college chemistry curricula across institution types because of the well-documented positive impacts they have on student learning and retention.^1−3^ However, fewer studies have focused on the impact, both positive and negative, of CUREs on faculty.^4−6^ When developing CUREs, faculty can either design a CURE based on their own independent research program, design a CURE unrelated to their research, participate in a multisite CURE network, such as the Malate Dehydrogenase CURE Community,^7^ or utilize previously developed CUREs from pedagogical journals or online repositories such as CUREnet,^8^ all of which will provide the same benefit for students. Utilizing faculty research for CUREs is generally encouraged^1,2,9^ because the benefits, which include being able to pilot new projects, generate publishable data, and get preliminary results for grant proposals, help justify the high faculty workload and financial cost associated with CURE development that can deter faculty from implementing CUREs in their courses.^4−6^ However, few studies have discussed how to specifically achieve the stated benefits for faculty related to data generation and publication when designing CUREs or the rate at which these benefits occur in general. Therefore, a set of best practices that maximize the research benefits of CUREs for faculty is needed.

This paper discusses the iterative development of an upper-level, semester-long, interdisciplinary CURE that did result in preliminary data for a grant proposal and a scientific publication. It also examines the rates and challenges associated with publishing CURE data based on a national survey of chemistry faculty who have taught CUREs across institution types. From these, a series of best practices to consider when developing CUREs so as to increase benefits for faculty while maintaining the well-documented benefits for student benefits have been developed.

## UNC Asheville Drug Discovery Project Laboratory

In 2015–2016, the Department of Chemistry and Biochemistry at the University of North Carolina Asheville revised its curriculum, and as part of that revision, all upper-level (3rd and 4th year) discipline-specific laboratories were replaced with research-based Interdisciplinary Chemistry Project Laboratories (ICPLs) that build off of at least two subdisciplines in chemistry.^10^ The goal of this change was to transform all advanced laboratory courses into CUREs that utilize foundational knowledge from multiple subdisciplines of chemistry to understand or interrogate complex chemical problems. Integration of chemistry subdisciplines into a single laboratory course has been shown to increase student understanding of the foundational concepts, increase student interaction with instrumentation and cutting-edge methods, improve written and oral communication skills, reduce barriers to accessing undergraduate research, and promote student retention in the major. However, these types of integrated laboratories suffer from the same drawbacks as other CUREs, such as increased faculty workload for both development and execution, high cost of implementation, and lack of administrative support.^11−15^ To reduce the challenges of implementation, the department chose to utilize team-teaching to reduce workload and foster collaborations within the department. Additionally, ICPL projects were developed based on individual research programs with the hopes of generating usable research through these courses.

One ICPL that was developed by the authors, which integrates organic chemistry, biochemistry, and computational chemistry, is a semester-long CURE focused on antibiotic drug discovery. Specifically, the goal of this ICPL is to generate small molecule inhibitors of *Pseudomonas aeruginosa* (*PA*) ATP synthase, and during the ICPL students rationally design inhibitors using computational docking, synthesize them, and then evaluate them in *in vitro* ATP synthesis inhibition assays and cell death assays against PA. This project was based on a collaboration between the Wolfe and Steed laboratories that was in its initial stages and had the goals of generating preliminary data for a grant proposal and, ideally, research publications with students from the course as lead authors. Additionally, development of this course was supported through an external award (Research Corporation for Science Advancement Cottrell Scholar Award), which greatly reduced the cost barriers for course implementation. Below the course structure is detailed, and the changes that have been made over 4 consecutive fall semesters (Fall 2019 to Fall 2022) to improve both student outcomes and research productivity are highlighted. To date, the ICPL has resulted in one research publication^16^ with 11 undergraduate coauthors.

### Laboratory Design

**Figure 1:**
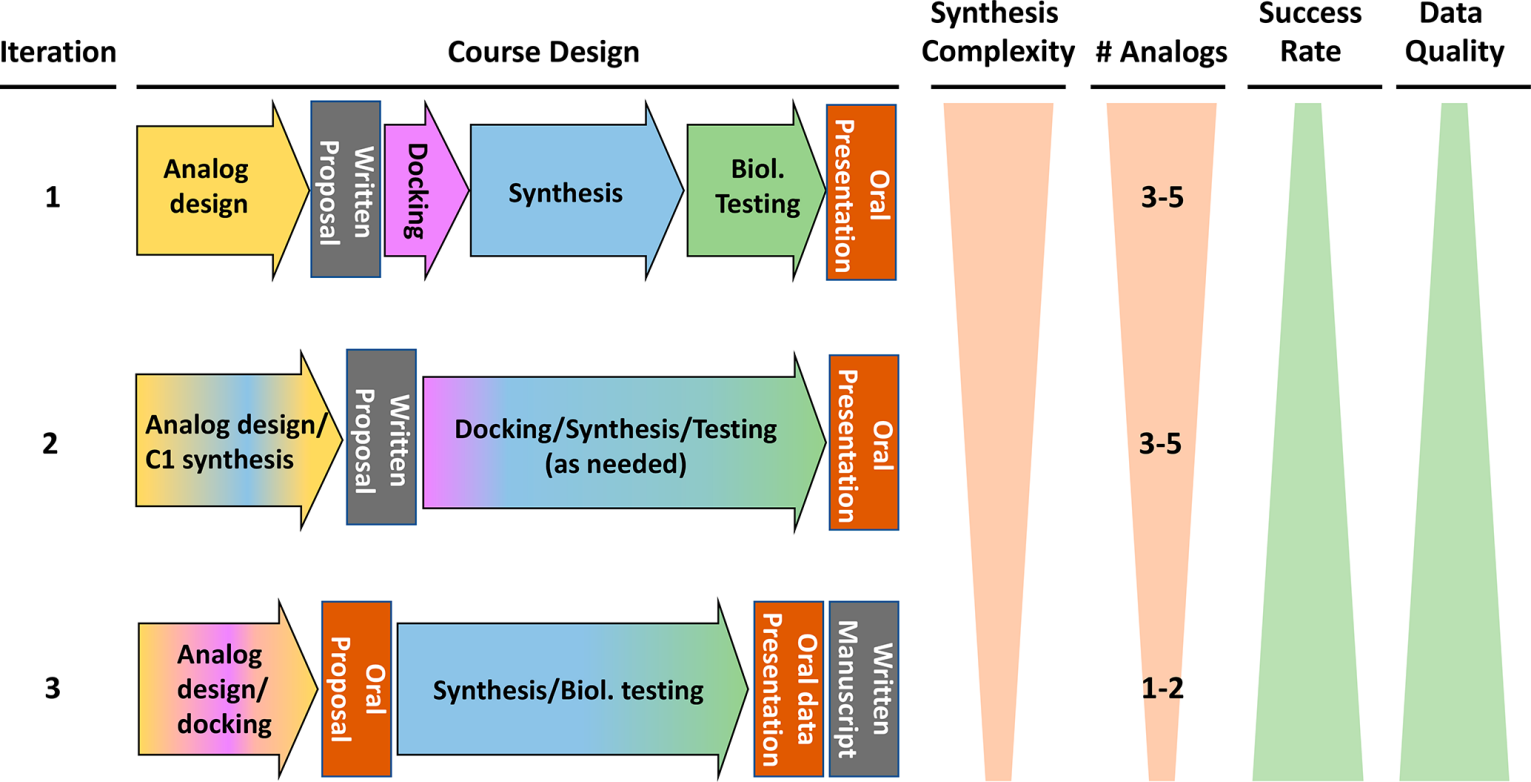
Iterative design of the Drug Discovery ICPL. Course elements are color-coded in I1, and mixed colors within a block indicate a combination of course elements.

Since Fall 2019, the Drug Discovery ICPL has had 3 major iterations (I1, I2, and I3), as shown in Figure 1. Broadly, these iterations have transitioned the course from an exploratory to a focused compound design strategy while maintaining the same Student Learning Outcomes (SLOs), which are that students will:

1. Use their knowledge of molecular structure, electronics, and protein–target interactions to develop a library of molecules based on the desired biological target.
2. Synthesize and characterize complex organic molecules using common organic chemistry techniques, NMR spectroscopy, IR spectroscopy, and mass spectrometry.
3. Assay synthesized and control compounds using common biochemical techniques.
4. Analyze and relate data from multiple experiments to draw informed conclusions.
5. Write a clear, concise, and persuasive research proposal (I1/I2) or manuscript (I3) using their expertise in organic chemistry, biochemistry, and computational chemistry.
6. Defend or modify their hypotheses orally based on data they obtain.

The 2-credit hour course typically has between 16 and 20 junior/senior level students enrolled who work in teams of 2 and meet for 3 h on two consecutive days a week for 15 weeks. In all iterations of the course, the major assessments included an oral presentation, a written assignment, and an electronic notebook. Finally, at the beginning of each semester, mini-lectures on ATP synthase structure and function, medicinal chemistry and structure activity relationship studies, and computational chemistry are given to prepare students for the project.

In the first iteration (I1) of the course, students were asked to design, synthesize, and biochemically interrogate 3–5 quinoline analogs. The semester was divided into discrete phases that mimic how a principal investigator (PI) approaches research projects (idea generation, proposal, revision, execution, presentation of results), as seen in Figure 1. During the first 4 weeks of the course, while also attending mini-lectures, students searched the literature and wrote a 5-page National Institute of Health style grant proposal detailing their objectives and hypothesis (SLOs 1, 5, and 6). Students then defended their proposals orally to a group of their peers who acted as grant reviewers (SLO 6). After the proposal review panels and approval by faculty, students spent 2 weeks docking their proposed analogs using AutoDoc Vina^17,18^ (SLO 1). During the 2 weeks of docking, faculty ordered chemical reagents needed for the synthesis phase. The next 6 weeks were dedicated to synthesis and spectroscopic characterization of analogs (SLO 2). Two weeks were then dedicated to biological testing, which consisted of a broth microdilution bacterial cell death assay against *PA* and an *in vitro* ATP synthase inhibition assay (SLO 3). Finally, students prepared oral presentations of their results and presented them to the class as a final exam (SLO 4). During the Q/A session of the presentations, faculty asked probing questions about data and design that were used to assess student understanding (SLO 6).

In I1, students had complete intellectual freedom in analog design, which led to relatively complex analogs being proposed. Over 50 analogs across 13 groups were proposed using a variety of multistep synthetic approaches. Due to this complexity, synthetic success and compound characterization were limited and having discrete phases prevented students from being able to troubleshoot their designs when synthetic challenges occurred. Therefore, few compounds (<10) advanced as far as biological evaluation and only 2 compounds were able to be published.^16^ Additionally, since students proposed their own mini-structure–activity relationship (SAR) studies, even the compounds that were generated were not cohesive enough to draw conclusions. Finally, since the writing assessment was in the form of a proposal, no manuscript preparation occurred during the course.

To address the challenges in I1, two major modifications were made for I2. Analog design parameters were limited to modification of only one position on the quinoline core (aldehyde chemistry), but any modification could be proposed (target was still 3–5 analogs per group), and the phases were blended so that redesign could occur if needed. This iteration produced a larger number of analogs that were successfully synthesized, characterized, and evaluated, with 8 student proposed compounds being included in the publication^16^ (while still meeting all SLOs and promoting student intellectual creativity). This iteration still did not have a written assessment that could be translated into a manuscript. Limiting the area of modification allowed the class to produce a more cohesive SAR study, which was able to be successfully published. However, preparation of the manuscript required the PIs to synthesize additional compounds, resynthesize and fully characterize student compounds, and conduct numerous control experiments, which in total took approximately 8 months to complete and was not part of the course teaching workload.

Using the lessons learned from I1, I2, and publishing the first manuscript with student data, I3 was designed to lower the barriers to publication including: (1) removing the need for PI and research students to repeat synthesis, characterization, and assays to obtain more synthesized product and produce higher quality data and (2) removing the need for PI to write a manuscript from scratch, while facilitating the production of publication-quality data and still allowing student independence to make a genuine contribution to the research. The students were also directly told at the beginning of the course that the goal was to generate a high-quality research publication and used that as the rationale for the course design and the required assessments. This goal not only provided context but also got students more excited about the project.

In I3, to further minimize synthetic hurdles, the analog design was narrowed to be focused on the initial published SAR study and to rely on a single robust synthetic reaction (reductive amination of an aldehyde). The computational docking process was also embedded into the analog design phase and used the Schrödinger “Teaching with Schrödinger”^19^ software to increase docking efficiency. Using their docking results and the literature, students then proposed 3–5 analogs, knowing that they would only target 1–2 synthetically following proposal review. Limiting the number of target analogs allowed for more time for troubleshooting, spectroscopic characterization, and biochemical evaluation, including control assays, within a one-semester time frame without diminishing student intellectual freedom. Finally, the assignment order was inverted so that students orally proposed synthetic targets and concluded by writing a manuscript to summarize SAR data from the entire class, which better aligned with what the actual publication would entail. Whereas I1/I2 aligned with the PI approach, this iteration more closely aligned with how members of a research team approach a project: pitching several ideas then choosing a target to pursue, reporting data in a research-group-like meeting, combining data into one SAR interpretation, and drafting a manuscript. I3 provided the highest quality data of the 3 iterations, with 8 of 9 groups successfully synthesizing, characterizing, and evaluating their chosen compounds, as well as initial manuscript drafts, despite being a smaller SAR study overall. This work, generated in Fall 2022, is currently being prepared for publication.

## CURE Survey

In addition to evaluating the previously described experiences in developing a CURE that is highly motivating and beneficial for both students and faculty, it is important to also understand other faculty’s experiences and determine whether there were common factors that promote or inhibit the successful publication of data generated from CUREs. To that end, a national survey of chemistry faculty who teach CUREs at public and private primarily undergraduate institutions (PUIs, Bachelor/Masters granting only) and public and private Research Intensive (R1, Ph.D. granting) institutions was conducted, and the results are detailed below.

To recruit study participants, the authors sent individual emails with the survey to faculty (>475) from the Research Corporation for Science Advancement Cottrell Scholar Network, which has a demonstrated history of members who engage in CUREs,^1,2^ faculty (>50) in Chemistry/Biochemistry Departments at institutions in the Council of Public Liberal Arts Colleges, and faculty who the authors believed matched our study criteria, i.e. faculty with active independent research programs who teach CUREs. We also encouraged faculty to broadly share the survey with colleagues. Through this solicitation a total of 70 responses from faculty within the United States, with 51 of those responses self-identifying as being in Chemistry or a related subdiscipline, were received. Although not explicitly excluded from the survey, no responses were received from faculty participating in a multisite CURE network or faculty outside of the United States; therefore, all results are from the perspective of independent research at US higher education institutions.

The survey (see the Supporting Information) was designed to gather basic descriptive data on faculty participants, including institution name, academic discipline, academic position/rank, and course type/level. The survey also gathered data on CURE design, including whether the CURE was individual or team-taught, whether the CURE was experimental, computational or a mixture of both, and whether the CURE was related to the faculty’s primary research. For CUREs that resulted in peer-reviewed scientific (nonpedagogical) publications, additional information on the time and resources required to successfully publish was gathered. A single open-ended question on the challenges associated with publishing data generated from CUREs was also posed, but no individual or follow-up interviews were conducted. The University of North Carolina Asheville Institutional Review Board approved this study (1937536-1).

As stated, 51 faculty in Chemistry or related subdisciplines from 37 institutions completed the survey. Of the 51 faculty, 40 self-identified as having taught/developed a CURE as part of a course. Only 10 of those faculty reported that data generated from their CURE were published as part of a scientific peer-reviewed journal article; however, 21 of the faculty who did not publish said that they hoped to in the future. As seen in Table 1, faculty at private PUIs had the highest rate of publication, and faculty at public PUIs had the lowest rate of publication, which was found to be statistically significant at a 95% confidence interval via two-tailed *t* test. Both public and private R1 institutions had lower publication rates compared to private PUIs as well, but due to sample size, the statistical significance was only at a 88% confidence interval via two-tailed *t* test. Unsurprisingly, more senior faculty (associate and full professors) have taught CUREs than faculty at the rank of assistant professor, but low response numbers make quantitative analysis of publication rates based on rank inconclusive. Of the CUREs taught, all but 4 were laboratory CUREs, and of the laboratory CUREs, publication rates were similar for lower-level and upper-level courses. Interestingly, all of the CUREs that resulted in publications were either entirely or partially experimental. However, a larger survey of computational (theoretical) only CUREs would need to be conducted to further assess this observation. CUREs that were taught by individual faculty versus those that were team-taught resulted in a similar rate of publication in general. PUIs were more likely to use a team-teaching approach compared to R1s and those PUI faculty that engaged in team-teaching saw a slightly higher rate of publication. Finally, the majority of CUREs were based on the faculty’s independent research programs, which resulted in a slightly higher publication rate.

**Table 1:** Results from Faculty Survey of CUREs (*n* = 40)

Question	Have not published	Have published [% total]
Q1. Institution Type		
Private PUI	7	6 [46%]
Public PUI	9	1 [10%]
Private R1	3	1 [25%]
Public R1	11	2 [15%]
Q2. Academic Rank		
Assistant Professor	2	2 [50%]
Associate Professor	17	2 [11%]
Professor	11	6 [35%]
Q3. CURE: Course and Level		
Lower-Level Lecture	2	0 [0%]
Lower-Level Lab	7	5 [42%]
Upper-Level Lecture	2	0 [0%]
Upper-Level Lab	19	5 [21%]
Q4. Was your CURE team-taught?		
Yes^a^	8	4 [33%]
No	22	6 [21%]
Q5. CURE Research type		
Experimental^b^	21	9 [30%]
Computational	3	0 [0%]
Both	6	1 [14%]
Q6. Was your CURE related to your research?		
Yes	25	9 [26%]
No	5	1 [17%]

a Of the 12 total team-taught CUREs, 10 were at PUIs.

b Experimental laboratories were defined as having a “wet-lab” component.

To probe how much additional time and effort is required to successfully publish data generated during a CURE, faculty respondents who published CURE data were asked three additional questions as seen in Table 2. Although the number surveyed is low, most publications included multiple semesters of CURE data and all required follow-up work to be completed by either the faculty or other researchers not associated with the course. Additionally, publication did not occur until 1–3 years after the CURE data were initially generated. The most common challenges associated with publishing CURE data based on the open-ended survey question included:

1. Undergraduate student turnover, which impacted both the data generation and manuscript writing.
2. Time to analyze data and write manuscripts.
3. Necessity (and time needed) to reinforce results, especially for compound/material characterization, for publication quality.

**Table 2:** Results from Faculty Survey of CUREs Who Published (*n* = 10)

Question	Responses
Q1. How many semesters of the CURE were needed to gather the data that was published?	
1 semester	2
2 semesters	3
≥3 semesters	5
Q2. How long after you finished collecting the data in the CURE did you publish the results?	
<1 year	0
1–2 years	5
2–3 years	5
Q3. Did you or your research students not enrolled in the CURE have to supplement the CURE data to make the results publishable (i.e., had to gather more data, rerun experiments/controls, etc.)?	
Yes	10
No	0

These findings concur with the authors’ CURE experience as described above and are significant because they highlight that even well-developed CUREs that produce high-quality data need follow-up effort from the faculty/other researchers.

## Maximizing CURE Impact on Faculty

While the positive impact on faculty research through data generation and publication is frequently used as an additional reason, beyond benefit to the students, for why faculty should develop CUREs, this impact is not guaranteed. Therefore, when developing CUREs that are to be used to advance independent research programs, faculty should be aware of the additional resources that will be required, such as additional time, supplies, and personnel for follow up and confirmation experiments, and should design their courses to facilitate high-quality record keeping and data generation. Based on the authors experiences developing a research productive CURE at a public PUI and the survey results, a list of tips and considerations for faculty, especially those at public PUIs who typically have higher teaching loads and lower resources, who hope to reap the research benefits of CURE development have been devised as seen in Table 3.

**Table 3:** Tips for Increasing Publication Rates from CUREs

Challenges	Tips (Challenges addressed)
1. Lack of faculty time to develop and implement CUREs	• Team teach CUREs to help reduce individual faculty workload, foster research collaboration, and provide students an opportunity to engage in interdisciplinary research (1 and 4).
2. Lack of cohesive data to generate a publishable story.	• Utilize experimental design guidelines that result in a useful class-wide data set at the end of the course (2–4).
3. Lack of quality student generated data.	• Narrow the focus of student projects to generate higher quality data during the course, which reduces follow-up work needed to publish (2–4).
4. High faculty effort needed after the course concludes to prepare data for publication and write the manuscript.	• Build in time to troubleshoot and overcome challenges, which provides students an opportunity to get creative and results in more high-quality data generation (3–4).
	• Incorporate manuscript preparation into the course assessments, which reduces barriers to publication and provides students an opportunity to understand what is required in a scientific manuscript (4).

Additionally, based on the experiences described and the survey results, some considerations for faculty who are developing research productive CUREs are

1. Administrative support is often needed regarding scheduling, resources, and team teaching.
2. Initial startup costs should be planned for.
3. Multiple semesters are often needed to generate publishable data and some follow-up work will need to occur but can be minimized through course design.
4. Student buy-in is essential.

In conclusion, this work has demonstrated that while CUREs can be used to initiate research projects and gather preliminary data, there are significant barriers to developing a CURE that can generate publication quality research. However, these barriers can be overcome through careful planning and execution. Ultimately, unlocking the research power of CUREs will allow for increased research productivity, especially at PUIs.

## Supporting Information Available

CURE Survey Instrument (PDF, DOCX)
