# Supplementary material for: Generating Publishable Data from Course-Based Undergraduate Research Experiences in Chemistry
Source: J Chem Educ. 2023 Aug 10;100(9):3419–24. doi: 10.1021/acs.jchemed.3c00354 (PMC10501119; doi:10.1021/acs.jchemed.3c00354)
Supplement: Supplementary file 2 — ed3c00354_si_002.docx [file ed3c00354_si_002.docx]

Supporting Information: Generating Publishable Data from Course-Based Undergraduate Research Experiences in Chemistry

Amanda L. Wolfe^1^* and P. Ryan Steed^1^

^1^Department of Chemistry and Biochemistry, University of North Carolina Asheville, One University Heights, Asheville, North Carolina, 28804, United States

*corresponding author email: awolfe@unca.edu

**Table of Contents**

CURE Faculty Survey Instrument Questions S2

**CURE Faculty Survey Instrument Questions.**

**Survey Flow**

Block: Demographic Questions (5 Questions)

Standard: CURE Yes/No (1 Question)

Branch: New Branch

If

If Have you taught a CURE (Course-based Undergraduate Research Experience) before? No Is Selected

EndSurvey:

Branch: New Branch

If

If Have you taught a CURE (Course-based Undergraduate Research Experience) before? Yes Is Selected

Block: Cure Questions (5 Questions)

Branch: New Branch

If

If Did you publish results from data generated in your CURE? Yes Is Selected

Block: CURE Details (6 Questions)

Standard: End (1 Question)

EndSurvey:

Branch: New Branch

If

If Did you publish results from data generated in your CURE? No Is Selected

Standard: End (1 Question)

EndSurvey:

Branch: New Branch

If

If Did you publish results from data generated in your CURE? Not yet but I hope to in the future Is Selected

Standard: End (1 Question)

EndSurvey:

| Page Break |  |
| --- | --- |

Start of Block: Demographic Questions

Q1 What is your email? (this information will not be distributed)

________________________________________________________________

Q2 What is your academic rank?

- Assistant Professor (1)
- Associate Professor (2)
- Professor (3)
- Lecturer (4)
- Other (5) __________________________________________________

Q2 What institution are you affiliated with?

________________________________________________________________

Q3 What type of institution is your institution (select all that apply).

- Research Intensive (1)
- PUI (2)
- PhD granting Institution (3)
- Bachelors/Masters only granting Institution (4)
- Private (5)
- Public (6)

Q5 What department are you in?

- Chemistry and/or Biochemistry (1)
- Physics (2)
- Astronomy (3)
- Other (4) __________________________________________________

End of Block: Demographic Questions

Start of Block: CURE Yes/No

Q1 Have you taught a CURE (Course-based Undergraduate Research Experience) before?

- No (1)
- Yes (2)

End of Block: CURE Yes/No

Start of Block: Cure Questions

Q7 Which type of course was your CURE in?

- Lower level (1st or 2nd year) lecture (1)
- Lower level (1st or 2nd year) laboratory (2)
- Upper level (3rd or 4th year) lecture (3)
- Upper level (3rd or 4th year) laboratory (4)

Q18 Was your CURE team taught (i.e. taught by more than one faculty per semester)?

- No (1)
- Yes (2)

Q9 Was your CURE related to your own research projects being performed by people in your research laboratory?

- No (1)
- Yes (2)

Q21 Was your CURE 

- Computational/data analysis (1)
- Experimental (2)
- Combination of both computational and experimental (3)
- Other (4) __________________________________________________

Q10 Did you publish results from data generated in your CURE?

- No (1)
- Not yet but I hope to in the future (2)
- Yes (3)

End of Block: Cure Questions

Start of Block: CURE Details

Q11 Where did you publish results of your CURE?

- Pedagogical Peer Reviewed Journal (1)
- Scientific Peer Reviewed Journal (2)
- Both (3)
- Other- Please explain (4) __________________________________________________

Q12 What is/are the citation(s) for your CURE related publication?

________________________________________________________________

Q15 How many semesters of the CURE were needed to gather the data that was published?

- 1 semester (1)
- 2 semesters (2)
- 3 semesters (3)
- more than 3 semesters (4)

Q13 How long after you finished collecting the data in the CURE did you publish the results?

- Less than 1 year (1)
- 1-2 years (2)
- 2-3 years (3)
- More than 3 years (4)

Q14 Did you or your research students not enrolled in the CURE have to supplement the CURE data to make the results publishable (i.e. had to gather more data, re-run experiments/controls, etc.)?

- No (1)
- Yes (2)

Q17 What was the biggest challenge you faced while working to get science generated in the CURE published?

________________________________________________________________

________________________________________________________________

________________________________________________________________

________________________________________________________________

________________________________________________________________

End of Block: CURE Details

Start of Block: End

Q20 Thank you for your time filling out this survey. Would you be open to being contacted to discuss your experiences in CURE development/publication more in depth in the future?

- No (1)
- Maybe - Depends on the amount of time it would require (2)
- Yes (3)

End of Block: End
